# Voluntary salt-reduction target setting by large food companies: a scoping review and questionnaire survey

**DOI:** 10.1038/s41440-025-02433-3

**Published:** 2025-11-14

**Authors:** Miwa Yamaguchi, Ikuko Kashino, Katsuyuki Miura, Nobuo Nishi, Nayu Ikeda

**Affiliations:** 1 National Institute of Health and Nutrition, National Institutes of Biomedical Innovation, Health and Nutrition, Osaka, Japan; 2 College of Gastronomy Management, Ritsumeikan University, Shiga, Japan; 3 Department of Nutrition, Koshien University, Hyogo, Japan; 4 NCD Epidemiology Research Center, Shiga University of Medical Science, Shiga, Japan; 5 Japanese Society of Hypertension, Tokyo, Japan; 6 Graduate School of Public Health, St Luke’s International University, Tokyo, Japan

**Keywords:** Corporate targets, Food industry, Product reformulation, Salt reduction, Scoping review

## Abstract

Dietary salt reduction is essential for the prevention of hypertension. However, food companies often lack reference information for setting appropriate salt-reduction targets. This study aimed to clarify how leading food companies in high-income countries outside Japan establish salt-reduction targets for product reformulation. A scoping review was conducted using PubMed, Google Scholar, and corporate websites. A questionnaire survey was also administered via email to companies with recognized good practices from January to March 2024. The study analyzed the scope of targeted products, salt (including sodium) content criteria, and achievement years, as well as incentives and challenges related to salt reduction. A total of 24 companies and two trade associations were included in the analysis. Among the 40 identified targets, 55% were based solely on salt, while the remaining included multiple nutrients. Regarding reformulation efforts, 68% of targets specified product quantities or proportions, and 55% committed to salt reduction in 33–100% of their products. Furthermore, 65% of the targets set salt criteria by food category; 80% used company-defined salt criteria, while the remaining used government-proposed benchmarks. The median implementation term was five years, with 65% of targets set to be achieved by 2025. Environmental, social, and governance investments were recognized as key incentives. To address reformulation challenges, stepwise approaches based on stakeholder cooperation were proposed. Overall, salt-reduction targets were set within each company’s feasible range, considering government policies and global trends. These findings provide a foundation for promoting company-led initiatives to reduce salt intake and contribute to hypertension prevention.

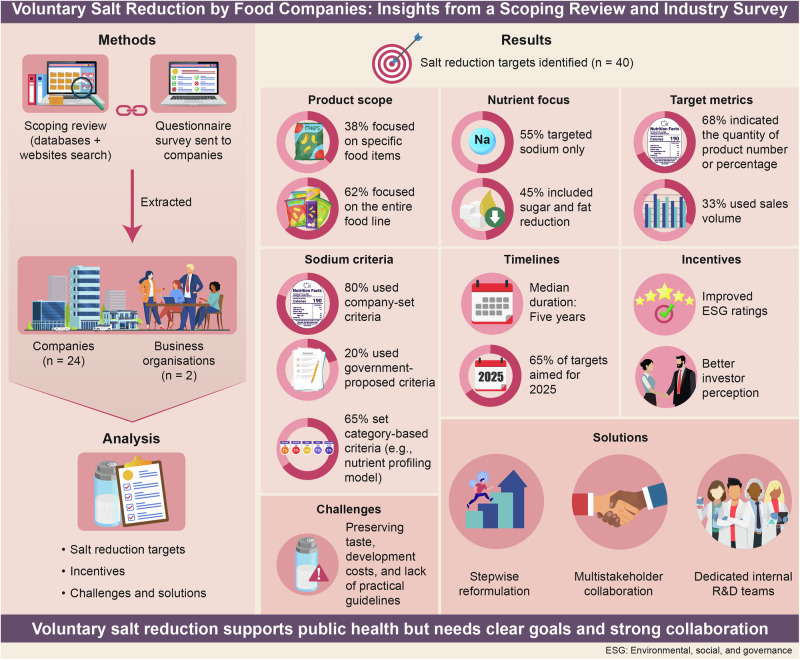

## Introduction

Dietary salt (including sodium) reduction is a cost-effective strategy to improve population health and prevent non-communicable diseases [1]. Accordingly, the World Health Organization (WHO) recommends a daily salt intake target of 5 g [1], and all member states of the WHO have committed to reducing population salt/sodium intake by 30% by 2025 [2]. However, this target has not been achieved even in high-income countries [3]. According to a systematic review, 96 countries had developed national salt-reduction strategies by 2019, which is three times the number reported in 2010 [4, 5]. These programs often involve collaboration with the food industry in the context of product reformulation, setting salt content targets for specific foods, and front-of-pack labeling [6]. The WHO assessed national efforts to reduce sodium intake through government policies and other initiatives [7]. Although mandatory government-led policies are generally more effective, only 5% of member states have implemented such policies. Therefore, it remains essential to promote voluntary actions alongside government initiatives.

As product reformulation is a key strategy for reducing population salt intake [7], voluntary reformulation through government-industry collaboration has been implemented in many countries. An Australian study modeled the potential health impact of achieving nationally set sodium reformulation targets [6]; the findings indicate that full compliance could prevent approximately 1,920 cases of cardiovascular disease, chronic A practical example is provided in the United Kingdom (UK): kidney disease, and stomach cancer, as well as avert 510 deaths and save approximately 7240 health-adjusted life years annually. A practical example is provided in the United Kingdom (UK): salt-reduction targets were set for more than 85 food categories between 2003 and 2011 [8]. Collaboration with the food industry resulted in a 20–50% reduction in the national average salt intake from 9.5 g/day to 8.1 g/day [8]. Similarly, the national food agency in Spain coordinated salt reduction initiatives targeting potato chips, savory snacks, meat products, and bread between 2018 and 2020, in cooperation with over 500 companies [8].

Despite these efforts, recent salt-reduction activities have encountered several challenges. While benchmarks based on actual corporate goals could aid decision-making, there is currently no comprehensive report that identifies feasible targets set by companies. Previous studies have examined national initiatives [5] and global salt reduction in packaged foods [9–11]; however, company-level goal-setting remains unexplored. Furthermore, while governments and the WHO have published several salt-reduction guidelines [12–15], the clarity and specificity of corporate goal-setting remain insufficient. In Japan, the Initiative for a Healthy and Sustainable Food Environment—a multisectoral and multistakeholder strategy—was launched by government sectors in 2022 [16]. However, to the best of our knowledge, efforts to support company-set salt reduction are still under development, and only a limited number of companies have disclosed salt reduction as an official commitment. At the global level, the Access to Nutrition Initiative (ATNI) has monitored the overall “healthiest” portfolios and the “healthiest” products within categories [17]. Strengthening transparent goal-setting for salt reduction would further contribute to such global health initiatives. In addition, corporate annual reports rarely disclose the practical challenges of reformulation, even though they are common. Companies with good practices could share these barriers to help others find solutions.

This study aimed to review voluntary salt-reduction targets through product reformulation by large food companies with recognized good practices in high-income countries outside Japan. To complement information on reformulation strategies, it also examined the incentives and challenges companies faced to assess the feasibility and value of voluntary salt-reduction efforts. Moreover, this study examines strategies for the external communication of goals and for setting achievable targets, serving as a reference for companies when formulating future salt-reduction objectives both in Japan and internationally.

Point of view

- Clinical relevance
  Voluntary salt reduction by food companies supports individuals in making healthier food choices that help reduce salt intake.
- Future direction
  Stronger collaboration between governments and food companies, through both policy-based and voluntary initiatives, can bridge the gap between clinical recommendations and the food supply.
- Consideration for the Asian population
  Given that high salt intake remains prevalent in Asia, collaborative efforts are essential to achieving effective reductions in population-level salt intake.

## Methods

### Design

A scoping review protocol was employed to (1) state the objectives and specific review question, (2) define the inclusion criteria, (3) search for relevant studies and documents, and (4) extract and chart the results [18]. This scoping review followed the guidelines outlined in the Preferred Reporting Items for Systematic reviews and Meta-Analyses extension for Scoping Reviews Checklist (Supplementary Table 1) [19].

The review question was as follows: *How do companies set reformulation goals aimed at salt reduction (including sodium reduction)?* To address this question, we conducted a scoping review to map the goal-setting strategies for salt reduction through product reformulation. We also examined the incentives and challenges associated with reformulation.

### Inclusion criteria

Data were collected through three steps: (1) a literature search of research databases, (2) a website search of the official websites of food companies and relevant organizations, and (3) a cross-sectional questionnaire survey distributed to selected food companies. The inclusion criteria for the literature and website searches were as follows: (1) voluntary salt reduction through product reformulation, (2) companies headquartered or operating in high-income countries outside Japan that promote voluntary salt reduction [7] (“high-income countries” were determined according to the income classification published by the World Bank [20]), (3) clearly defined salt-reduction goals through reformulation, (4) reformulation targeting the general population (not for nutritional treatment), and (5) descriptions written in English.

### Database search

PubMed and Google Scholar were used to search the literature. Systematic keywords were developed based on the research question: “salt,” “company,” and “voluntary” (Supplementary Table 2). To identify company-specific reformulation cases, the term “case study” was added to the search. Two researchers (M.Y. and N.I.) independently conducted the search between February 8 and 26, 2024.

### Company website search

Potential companies were identified using four official websites that provide information on voluntary salt-reduction efforts by food companies: (1) ATNI, (2) Global Nutrition Report (GNR), (3) International Food & Beverage Alliance (IFBA), and (4) World Action on Salt, Sugar and Health (WASSH). The GNR, a multi-stakeholder initiative, monitors corporate commitments to improving global nutrition [21]. WASSH is a global health initiative focused on reducing salt, sugar, and unhealthy fat intake [22]. It also disseminates research on the health risks of excessive salt consumption. Keyword searches were conducted on these websites using terms such as “salt,” “reduction,” “commitment,” “report,” “target,” and “nutrition profile,” including their synonyms. Once potential companies were identified, information on salt-reduction initiatives was gathered from annual reports and relevant web pages. The search was independently performed by two trained technical assistants between January 15 and February 16, 2024.

### Questionnaire

A questionnaire was developed based on a relevant WHO publication [7], and an index was used to assess nutrition improvement efforts by food companies [23] (Supplementary Table 3). The questionnaire was pretested and finalized after incorporating feedback from four Japanese food companies. The questionnaire collected information on (1) target setting and efforts related to salt reduction through reformulation, (2) incentives from an environment, social, and governance (ESG) perspective, and (3) the challenges and potential solutions associated with salt reduction.

To identify food companies eligible for the survey, we obtained information through a research network comprising 19 researchers from 16 countries, two global organizations, and two authors cited in relevant studies. Based on this information, selected companies were invited by email to participate in the survey anonymously. The survey was conducted between January 19 and March 16, 2024, and followed the Consensus-Based Checklist for Reporting of Survey Studies guidelines (Supplementary Table 4) [24].

### Charting the data

The selection process aimed to identify companies with clearly defined salt-reduction targets through reformulation. The two researchers (MY and NI) independently conducted both the first and second screenings, blinded to each other’s decisions. No duplicates were identified between the two databases. Titles and abstracts were evaluated against the inclusion criteria during the first screening, while full texts were assessed during the second. Any disagreements were resolved through discussion to reach a consensus between the reviewers. For the website search, one researcher (M.Y.) screened the latest data, and the findings were validated in consultation with two trained technical assistants.

Data relevant to target setting were categorized as follows: (1) publication information from the literature (publication year, authors, title, and journal name) and from websites (year uploaded, name of company or organization, title, and URL), (2) company name, (3) target products for salt reduction, (4) salt criteria, and (5) achievement year. When the data on websites differed internally, the reviewer integrated them based on consistency and clarity. The final categorization was reviewed and discussed among one researcher (M.Y.) and two technical assistants to confirm the plausibility of the categories. All identified citations were exported and compiled in a Microsoft Excel spreadsheet. One researcher (M.Y.) extracted and summarized the data on the incentives and challenges related to salt reduction through reformulation from the questionnaire responses, verified by two other researchers (IK and NI).

### Synthesis of the results

The companies did not necessarily indicate a single target for salt reduction. Therefore, this study synthesized the various target units. The following target-setting components were identified: company, country, target products, salt criteria, and achievement year. Based on this information, target characteristics were quantified and classified into specific categories. These categories were determined through agreement among the authors taking into account the factors that companies should consider when formulating future salt-reduction objectives: target products (focused on specific products or entire product portfolio; quantified by product number (or proportion) or sales volume), target nutrients (only salt or salt in combination with other nutrients), criteria development (company-defined or government-proposed criteria), and salt criteria (classified by food category or a single criterion for all products). Food categories were classified using either a nutrient profile model or salt-only criterion. Nutrient profiling is “the science of classifying or ranking foods according to their nutritional composition for reasons related to preventing disease and promoting health” [25]. In addition to calculating the proportion of targets for each achievement year related to the total, the median, minimum, and maximum implementation term (years) were also determined. Incentives, challenges, and potential solutions were summarized. The Research Ethics Review Committee of the National Institute of Biomedical Innovation, Health, and Nutrition exempted this study from the “Ethical Guidelines for Medical and Biological Research Involving Human Subjects,” as no personal information was used.

## Results

### Identification and selection of companies

**Fig. 1:**
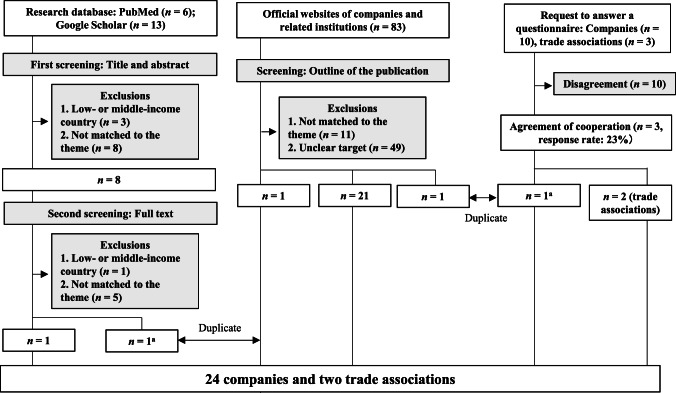
The flowchart shows the three information sources used to select the target companies: research databases, official websites of companies and related institutions, and questionnaire requests. ^a^Among the 23 companies extracted from official websites, two were found to be duplicates: one in the research database and the other in the questionnaire responses. Each company was counted only once in the analysis

In total, 19 studies (six from PubMed and 13 from Google Scholar) were identified through the research database search (Fig. 1). After an initial screening, 11 studies were excluded. Of the remaining eight, six were excluded for not meeting the eligibility criteria, leaving two studies for inclusion [26, 27], each describing one company. The website search identified 83 companies, with 23 retained after screening the outlines of the documents and webpages. For the questionnaire survey, 10 potential companies and three trade associations were identified through the research network. Responses were obtained from one company and two trade associations (response rate: 23%). Two companies identified through the website search were duplicates of those found in the database search and the questionnaire survey, respectively, and were merged accordingly. The salt-reduction targets of Lidl [26], which operates under the Schwarz Group, were included with those of the parent company. Williams et al. [27] reported on salt-reduction efforts by Kellogg Australia, which was treated separately from Kellanova. The Kellogg Company was split into two companies in 2023, one of which is Kellanova [28]. As a result, the final analysis included 24 companies and two trade associations.

### Characteristics of targets extracted from companies

In total, 40 targets were identified from 24 companies and two trade associations (Table 1). Regarding geographical scope, 68% (*n* = 27) were set at the global level [29–48], 25% (*n* = 10) at the European level [29, 48–52], 5% (*n* = 2) in Oceania [27, 53], and 3% (*n* = 1) in North America. The following eleven companies reported more than one target: Barilla [29], Grupo Bimbo [36], and Schwarz Group [48] each had three targets; Bel [30, 31], Ferrero [33], General Mills [35], Kellanova [37], Kraft Heinz [38, 39], Mars [42], Morrisons [51], and PepsiCo [45] each had two.

**Table 1:** Target settings for salt reduction through reformulation

Company	Target country	Product number or proportion	Sales volume	Sodium criteria	Implementation term
*Targets focused on salt*					
Aldi UK & Ireland	United Kingdom and Ireland	95% of products [54]	–	Salt reduction targets for 2024 [14, 49]	2020–2024
Barilla (Harrys)	France	100% of company bread [29]	–	Reduce salt level 1.2 ~ 1.1% [29]	2022–2023
Barilla (Harrys)	France	100% of company bread [29]	–	Reduce salt level 1.1% [29]	2022–2025
Barilla	Global	90% of company products [29]	–	0.5 g salt per portion [29]	2022–2030
Ferrero	Global	–	90% of the aggregated sales volume for the product portfolio (morning goods and biscuits) will meet the established category targets [33, 59]	Global Sodium Reduction Targets [33, 59]	2021– 2025
Ferrero	Global	–	At least 75% of the total sales volume of the product portfolio (morning goods and biscuits) will undergo sodium reduction [33, 59]	Global Sodium Reduction Targets [33, 59]	2021–2030
General Mills	Global	–	90% of the aggregated sales volume for the related product portfolio will meet the established category targets [35, 59]	Global Sodium Reduction Targets [35, 59]	2021–2025
General Mills	Global	–	At least 75% of the total sales volume of the product portfolio will undergo sodium reduction [35, 59]	Global Sodium Reduction Targets [35, 59]	2021–2030
Kellanova	Global	–	90% of the aggregated sales volume for the related product portfolio will meet the established category targets [37, 59]	Kellanova Global Nutrition Criteria [60]	2021–2025
Kellanova	Global	–	At least 75% of the total sales volume of the product portfolio will undergo sodium reduction [37, 59]	Kellanova Global Nutrition Criteria [60]	2021–2030
Kellogg Australia	Australia	12 cereal products [27]	−	Sodium contents <120 mg/100 g (for a low salt claim) and 400 mg/100 g [27]	1997 (one year)
Kraft Heinz	Global	One product each for barbecue sauce and salad dressing [38]	−	Reduce sodium by an additional 5% in products [38]	2022–2025
Lorenz	Global	Entire brand product portfolio (100%) [40]	–	Reduce the salt content in products by 15% [40]	2019–2025
McCain	Global	Potato and appetizer products (100%^a^) [41]	–	15% reduction in sodium (sales-weighted average) in products [41]	2017–2025
Mars	Global	Related product portfolio (100%^a^) [42]	–	5% reduction of sodium in product portfolio [42]	2021–2025
Morrisons	United Kingdom	84 food categories [51]	–	Salt reduction targets for 2024 [14, 51]	2020–2024
Nestlé	Global	–	90% of the aggregated sales volume for the related product portfolio will meet the established category targets [43]	Nestlé sodium reduction targets [43]	2021–2025
PepsiCo	Global	–	At least three-quarters (75%) of global foods portfolio sales volume [45]	Not exceed 1.3 mg/ c Calorie sodium [45]	2021–2025
PepsiCo	Global	–	At least three-quarters (75%) of global convenient foods portfolio volume [45]	PepsiCo global sodium targets [45]	2023–2030
Schwarz Group (Schwarz Produktion)	Germany	Baked goods (100% ^a^) [48]	–	Reduce the average salt content per kilogram by approximately 30% from the 2015 level [48]	2015–2025
Trade association A	Slovenia	Bakery products (100%^a^)	–	5% reduction of sodium in products	2019–2022
Trade association B	Chile	All bakery products (100%)	–	Sodium contents 400 mg/100 g in products [64]	2010–2014
*Targets focused on salt and other nutrients*					
Arnott’s Group	Australia and New Zealand	One-third of products [53]	–	Health Star Rating of 3.5 stars or more [53, 61]	2021–2025
Asda	United Kingdom	60% of own brand products [50]	–	UK Nutrient Profiling Model 2004/5 [50, 62]	2020–2024
Bel	Global	80% of children and family products portfolio [30]	–	Bel Nutri+ nutritional criteria [30]	2017–2025
Bel	Global	–	90% of sales of branded recipes targeting children and families [31]	Bel Nutri+ nutritional criteria [30, 31]	2017–2025
Bonduelle	Global	100% of own products [32]	–	Obtain a rating of A or B (top 2 rating of 5 points) on the Nutri-Score scale [32, 63]	2022–2025
FrieslandCampina	Global	–	At least 74% of sales volume [34]	FrieslandCampina Global Nutritional Standards [34, 55]	2020–2025
Grupo Bimbo	Global	100% of daily bread, buns, and breakfast portfolio [36]	–	Grupo Bimbo Nutritional Guidelines [36]	2022–2025
Grupo Bimbo	Global	100% of baking and snacks [36]	–	Grupo Bimbo Nutritional Guidelines [36]	2022–2030
Grupo Bimbo	Global	–	Top seller brands/products of the occasional consumption portfolio [36]	Grupo Bimbo Nutritional Guidelines [36]	2022–2025
Kraft Heinz	Global	85% of products [39]	–	The Kraft Heinz Global Nutrition Guidelines [39, 56]	2019–2025
Mars	Global	95% or more own products [42]	–	Mars Food Nutrition Criteria [42]	2021–2025
Morrisons	United Kingdom	65% of own-brand products [51]	–	Classify as non-HFSS (high fat sugar salt) according to UK Nutrient Profiling Model 2004/5 [51, 62]	2019–2025
Orkla	Global	Own products (100%^1^) [44]	–	15% less salt from the 2017 level [44]	2017–2025
Saputo	Global	84% of own products [46]	–	Saputo Nutrient Profiling Model [46, 57]	2023–2025
Schwarz Group (Lidl)	Global	Baked goods (100%^a^) [48]	–	Reduce the sales-weighted average salt and added sugar content by 20% from the 2015 level [48]	2015–2025
Schwarz Group (Kaufland)	Germany	500 private-label items [48]	–	Reduce the average salt, sugar, and/or fat content by 20% [48]	2015–2025
Tesco	United Kingdom and Ireland	–	Increase the proportion of healthy products (including low-salt products) in sales to 65% [52]	UK Nutrient Profiling Model 2004/5 [52, 62]	2021–2025
Unilever	Global	85% of own portfolio [47]	–	Unilever’s Science-based Nutrition Criteria [47, 58]	2022–2028

Target-setting components were classified by company, country, target products, salt/sodium criteria, and achievement year

^a^The numerical value was interpreted as the target for the entire brand product portfolio (100%) based on the context

### Target products

Of the 40 targets, 38% (*n* = 15) focused on specific product categories (Table 2), encompassing bread (*n* = 5) [29, 36], breakfast items including cereals (*n* = 4) [27, 33, 36], baked goods (*n* = 3) [36, 48], sweets and snacks (*n* = 3) [33, 36, 48], products for children and families (*n* = 2) [30, 31], potato and appetizer products (*n* = 1) [41], and sauces and dressings (*n* = 1) [38]. Regarding the target nutrients, 55% of targets (*n* = 22) focused solely on salt [27, 29, 33, 35, 37, 38, 40–43, 45, 51, 54], while 45% (*n* = 18) indicated other nutrients such as sugars and fats, in addition to salt [30–32, 34, 36, 39, 42, 44, 46–48, 50–53]. In terms of measurement units, 68% of the targets quantified salt reduction by product number [27, 29, 30, 32, 36, 38–42, 44, 46–48, 50, 51, 53, 54], and 33% by sales volume [31, 33–37, 43, 45, 52]. Four targets specified absolute numbers of products, such as 84 food categories recommended by the UK government (*n* = 1) [51, 54], 500 own-brand products (*n* = 1) [48], 12 cereal products (*n* = 1) [27], and one product each for barbecue sauce and salad dressing (*n* = 1) [38]. Among the 22 targets that specified a percentage of products, nine targeted 33–90% of own-brand products [30, 39, 42, 46, 47, 50, 51, 53], while the remaining 13 targeted all products (100%) [29, 32, 36, 40–42, 44, 48]. Most sales volume targets were expressed as percentages, with one specifically referring to top-selling own-brand products [36]. Furthermore, six targets were directly cited from the IFBA [33, 35, 37], and two others were related to IFBA targets [43, 45].

**Table 2:** Characteristics of target settings of salt reduction through reformulation

Characteristics	Target number, *n* (%)^a^
Specification of products	
Specific products	15 (38)
Entire products	25 (62)
Target included only salt or included other nutrients	
Only salt	22 (55)
Included other nutrients	18 (45)
Indication of target products	
Quantity	27 (68)
Sales volume	13 (33)
Criteria reference	
Company-set criteria	32 (80)
Government-proposed criteria	8 (20)
Type of criteria	
Criteria for each food category	26 (65)
Criteria for entire products	14 (35)
Type of food category criteria (26 targets)	
Nutrient profiling model	17 (43)
Salt-specific criteria	9 (23)

The 40 targets listed in Table 1 were quantified and classified into categories defined during the analysis

^a^The proportion was calculated based on the total number of targets (*N* = 40)

### Salt criteria

Of the salt reduction targets, 80% (*n* = 32) employed criteria defined by companies [30, 33–36, 39, 42, 43, 45–47, 55–60], while the remaining 20% (*n* = 8) adopted criteria proposed by government-related organizations [14, 32, 49–53, 61–64] (Table 2). Among the 32 targets using company-defined criteria, 18 focused on specific food categories [30, 33–36, 39, 42, 43, 45–47, 55–60], and 14 (35%) addressed the entire product portfolio [27, 29, 38, 40–42, 44, 45, 48]. Eleven food category criteria developed by companies referred to recommendations from the WHO and government-related organizations (Table 3). In addition to these references, one criterion was cited in a related study published by the company [65], and two criteria were introduced through expert insights [30, 57]. Government-proposed criteria were set exclusively for food categories.

**Table 3:** Criteria and the reference source for salt/sodium content in each product category used for target setting

Criteria for salt content	Reference source	Target number
Government-proposed criteria		
*Nutrient profile model*		
Health Star Rating system	Food Standards Australia New Zealand [61]	1
Nutri-Score	The European Consumer Organisation [63]	1
UK Nutrient Profiling Model 2004/5	Food Standards Agency [62]	3
*Salt content by product category*		
Salt Reduction Targets for 2024	Public Health England [14]	2
Salt content for bread	Government of Chile [64]	1
Company-defined criteria		
*Nutrient profiling models and associated functions*		
Bel Nutri+ nutritional criteria	WHO [82], Validation by 14 international experts [30]	2
FrieslandCampina Global Nutritional Standards	WHO, International Choices criteria [34, 55]	1
Grupo Bimbo Nutritional Guidelines	WHO [36]	3
The Kraft Heinz Global Nutrition Guidelines	WHO, European Food Safety Authority, National Academy of Medicine [52]	1
Kellanova Global Nutrition Criteria	Children’s Food and Beverage Advertising Initiative, IFBA [60]	2
Mars Food Nutrition Criteria	WHO, U.S. Department of Agriculture, U.S. Department of Health and Human Services, European Food Safety Authority, Food Standards Australia New Zealand, IFBA, Public Health England [42]	1
Saputo Nutrient Profiling Model	Global nutrient profiling models from governments, public health authorities, and industry bodies; consultations with leading nutrient profiling scientists [57]	1
Unilever’s Science-based Nutrition Criteria	WHO, research article [65, 83]	1
*Salt content by product category*		
Global Sodium Reduction Targets	WHO, IFBA [59]	4
Nestlé sodium reduction targets	WHO [43]	1
PepsiCo global sodium targets	WHO, U.S. Department of Agriculture, National Academy of Medicine, and other national public health authorities [45]	1

From the 40 targets listed in Table 1, the criteria and the reference source for salt content were identified, and the relevant targets were counted

*WHO* World Health Organization, *IFBA* International Food & Beverage Alliance

Of all targets, 65% (*n* = 26) were established based on salt-reduction criteria for each food category, while 35% (*n* = 14) were indicated as overall product criteria (Table 2). Among the 26 food category targets, the use of nutrient profiling models and associated functions was more common (43% of 40 targets, *n* = 17) [30, 32, 34, 36, 39, 42, 46, 47, 50–53, 55–58, 60–63] than criteria focusing solely on sodium content (23%, *n* = 9) [33, 35, 43, 45, 59, 64]. Of the five targets using nutrient profiling models based on government-proposed criteria, three [50–52] adopted the UK Nutrient Profiling Model 2004/5, developed by the UK government [62] (Table 3). The Health Star Rating System [61] and Nutri-Score [63], commonly used for front-of-package labeling, were used as criteria for each target. The Health Star Rating system, developed by Food Standards Australia New Zealand, provides a front-of-pack interpretive label ranging from 1/2 to 5 stars, with a higher number of stars reflecting greater nutritional quality [61]. Similarly, the French government introduced the Nutri-Score in 2017, which classifies foods on a five-color scale, from A (healthiest) to E (least healthy), and has been used to promote healthier consumer choices [66]. Both systems serve as tools to visualize overall nutritional quality and contribute to shaping healthier food environments. Beyond nutrient profiling, two targets [49, 51] adopted the salt-specific criterion “Salt-reduction targets for 2024” by the UK Public Health England [14]. Among the 18 targets using company-defined criteria, 12 used nutrient profiling models and associated functions [30, 34, 36, 39, 42, 46, 47, 55–58, 60], while six used sodium-specific criteria [33, 43, 45, 59].

Among the 14 targets addressing the entire product portfolio, six aimed to reduce salt content by 5–20% [38, 40, 42, 44, 48], two aimed to lower the sales-weighted average salt/sodium by 15–20% [41, 48], two targeted reducing salt concentration by 1.1–1.2% [29], and one aimed to reduce salt content per kilogram by 30% [48]. Targets expressed as sodium quantity set limits such as less than 120–400 mg sodium per 100 g (*n* = 1) [27], under 0.5 g salt per serving (*n* = 1) [29], and less than 1.3 mg sodium per calorie (*n* = 1) [45].

### Achievement year

Among the 40 targets, the median implementation term was five years, with a range of one to 11 years (Table 4). The most common target achievement year was 2025, accounting for 65% (*n* = 26) of the targets [29–42, 44–46, 48, 51–53, 59], followed by 2030 (15%, *n* = 6) [29, 33, 35–37, 45, 59].

**Table 4:** Implementation term and year for achieving targets

Implementation targets	Statistics
Implementation term, years: median (min., max.)	5 (1, 11)
Achievement year, *n* (%)	
2025	26 (65)
2030	6 (15)
2024	3 (8)
1997, 2014, 2022, 2023, 2028^a^	5 (13)

^a^These years were designated as achievement years for each of the 40 targets listed in Table 1

### Incentives and challenges in salt reduction through reformulation

The companies anticipated favorable evaluations from social and investor perspectives regarding ESG performance (Table 5). They expected increased investment driven by the ATNI, which assesses companies’ salt-reduction efforts. The companies reported challenges in achieving salt reduction while maintaining product taste. Economic challenges included rising development costs and the need to keep prices affordable during reformulation. Furthermore, the absence of established guidelines for salt-reduction efforts led to longer business planning times. As potential solutions, companies proposed creating a multi-stakeholder framework to support gradual salt reduction across the market and establishing internal teams specializing in salt reduction within research and development.

**Table 5:** Incentives, challenges, and resolutions in relation to salt reduction through reformulation

Topics	Responses
Incentives in terms of ESG	• Supporting consumers in making healthier food choices would contribute to sustainability efforts. • Given the emphasis that investors place on sodium reduction, the company committed to implementing a salt reduction strategy and reflected this commitment in its ESG report. • It is projected that investment would increase in response to the ATNI evaluation, including salt reduction initiatives.
Difficulties in implementing sodium-reduction actions and solutions	• The company halted a salt reduction initiative owing to difficulties encountered in product development and pricing. • Difficulties of salt reduction while maintaining consumer acceptance of the taste • Technical challenges including food safety • Cost of substitute ingredients • Time-consuming due to the lack of appropriate guidelines for salt reduction initiatives
Solutions to the difficulties	• A multi-stakeholder framework would ensure gradual salt reduction efforts across the market. • Establish a dedicated internal team to focus on salt reduction research and development.

Incentives, challenges, and resolutions were summarized based on responses obtained from one company and two trade associations

*ATNI* access to nutrition index, *ESG* environmental, social, and governance

## Discussion

This study examined voluntary salt reduction through reformulation in 24 companies and two trade associations. Of the 40 overall targets, 45% included nutrients other than salt. Results revealed that 80% of the targets set their own salt content criteria, often based on government or global recommendations, and 65% applied criteria by food category. The median implementation period was five years, with approximately 65% of targets to be achieved by 2025. While incentives from an ESG perspective were acknowledged in relation to product improvement, challenges such as maintaining original product taste and increased development costs were reported. To our knowledge, this is the first study to clarify how companies set targets for salt-reduction reformulation, revealing the actual status and their efforts.

### Characteristics of selected companies

Companies reporting salt-reduction goals were primarily based in European and Australian–New Zealand regions, where government policies actively promote salt reduction by companies. The European Salt Action Network, established with the endorsement of the WHO and support from the UK Food Standards Agency [67], facilitates coordination of salt-reduction programs across Europe. Similarly, the United States Department of Agriculture [68] and Food Standards Australia New Zealand [69] have also advanced initiatives toward salt reduction. In Japan as well, governmental sectors have been supporting companies in establishing a platform to promote salt reduction through the Initiative for a Healthy and Sustainable Food Environment [16]. This sustained initiative is expected to facilitate the broader development and availability of reduced-salt products, thereby contributing to population-wide salt reduction.

### Target setting for salt reduction

#### Target products

Approximately half of the targets involved reducing not only salt but also other nutrients such as fat and sugar. The UK has issued guidelines on restricting the promotion of high fat, sugar, and salt (HFSS) products [70]. Consequently, policies addressing excess intake of HFSS products have expanded, particularly in Western countries. Therefore, some companies have set product development goals aimed at reducing HFSS content.

Our results show that more targets quantified salt reduction by the number or percentage of products rather than by sales volume. Companies may have chosen the number or percentage of products as an understandable metric for stakeholders or consumers to demonstrate their commitment to salt reduction. However, using sales volume better reflects market trends. Six targets using sales volume were directly cited from the IFBA company alliance. The IFBA plans to review its targets in 2025, considering market fluctuations toward the 2030 target year [59]. Conducting intermediate evaluations could allow adjustments aligned with long-term goals.

Not all targets in this study aimed to cover 100% of a company’s products; some focused on subsets, such as 33–90% of products or 65–90% of sales volume. Product reformulation should be based on specific criteria. The US guidance on salt reduction primarily identifies foods using the following criteria: (1) contribution to national sodium intake; (2) total sodium content in the food; (3) technical feasibility of reducing sodium; and (4) compatibility with existing industry and regulatory categories [12]. These elements are critical for determining target categories and guiding salt-reduction efforts.

#### Salt criteria

Of the 40 overall targets, 80% used company-defined salt criteria, while 20% adopted criteria proposed by government-related organizations. The company-defined salt criteria often referenced recommendations from the WHO or from national government agencies. Companies likely adapted these recommendations to reflect domestic consumer preferences and market contexts. While the WHO recommends a global goal of 5 g salt per day [1], applying this directly as an initial target may be challenging for companies.

Salt content criteria were more frequently defined by nutrient profiling models than by salt-specific thresholds. Many companies used nutrient profiling models to assess the overall nutritional quality of products, including factors such as HFSS. Among the government-issued models, the UK Nutrient Profiling Model 2004/5 [62] was the most frequently used, particularly among UK-based companies. Detailed documentation available on the government website [71] may have facilitated its use. In addition, one target each used front-of-pack labeling systems such as the Health Star Rating System [61] from Food Standards Australia New Zealand and the Nutri-Score [63] developed by the European Consumer Organization. Both systems are widely recognized and used in international evaluations such as the ATNI [72], and may guide future salt reduction reformulation efforts.

Among the 18 targets based on the company-defined criteria, 12 used nutrient profiling models and six applied salt-specific thresholds. Two companies, each with one target, employed sodium-specific criteria instead of broader nutrient profiling models [73, 74]. Two IFBA member companies, each with two targets, supported the population-based salt-reduction strategy proposed by the WHO [59]. These approaches suggest strong motivation for salt reduction.

Regarding salt levels across all products, six targets set salt reduction rates of 5–20% over 4–11 years, while reductions of 15–20% were set for sales-weighted average salt/sodium over 9–11 years. For comparison, Canada’s guideline proposes a 25–30% reduction in sales-weighted average sodium content over seven years [75], and the UK achieved 20–50% reductions in various food categories over eight years [8]. These higher rates reflect differences in the context, including feasibility, consumer preferences, costs, and food safety considerations.

Two targets in this study used the sales-weighted salt/sodium average, which accounts for the sales volume of each product within a target food category [75]. This metric accords more weight to high-selling products, better reflecting actual consumption than a simple average. It is used in salt-reduction guidelines in the US, Canada, and the UK [12, 14, 75].

#### Target timeline

In this study, 65% of targets set 2025 as the achievement year, with a median implementation period of five years. National guidance on salt reduction for food companies proposes the following periods: 2.5 years (2021–2024) in the US [12], four years (2020–2024) in the UK [14], and five years (2020–2025) in Canada [75]. Bonduelle reported successful gradual reduction of salt content in its original recipes over the period from 2007 to 2022 [32]. Given these reports, an implementation period of five years appears reasonable for gradual salt reduction. Some companies may have chosen 2025 as the achievement year based on the timeline of the interim report for the Sustainable Development Goals [76]. The absence of targets beyond 2030 may be attributable to the 2030 deadline for achieving the Sustainable Development Goals, with companies potentially planning to set new targets after reviewing global trends.

#### Incentives and challenges in salt reduction through reformulation

Participating companies in the survey reported receiving incentives for their salt-reduction efforts in terms of ESG performance. For example, one company anticipated increased investment as a result of its evaluation by ATNI. Moreover, although it should be noted that this response came from only one company, several companies have also been recognized for their commitments and current achievements in relation to salt reduction as examples of good practice by ATNI [77–79]. Such efforts ultimately contribute to ESG performance. The health and nutrition category within ESG assessments has recently become a significant evaluation factor. A prominent tool to assess food and beverage companies’ performance related to nutrition improvement, including salt reduction, is the Global Index 2021 coordinated by ATNI [72]. Companies also reported challenges in maintaining product taste after salt reduction through reformulation. Developing low-salt products with acceptable taste requires considerable time, costs, and human resources. These challenges make it difficult to balance ambition with feasibility. Our findings suggest a need for multisectoral networks and more practical guidelines to assist companies in developing effective salt-reduction strategies. The WHO has cited the Islamic Republic of Iran and Japan as examples of good practice in promoting multisectoral action and supporting companies in implementing salt-reduction initiatives as part of non-communicable disease prevention [16]. Information sharing and dialog with government organizations can promote salt-reduction policies. Academic societies have also played an important role in multisectoral collaboration. For example, the Japanese Society of Hypertension has taken the lead in promoting salt-reduction initiatives in collaboration with companies and government authorities, through efforts such as developing a list of low-salt foods and encouraging the display of salt-equivalent content on food packaging [80]. Furthermore, guidelines are needed to set salt criteria, offer stepwise implementation frameworks, and facilitate networks that can support the development of salt-reduction initiatives.

### Strengths and limitations

This study conducted a scoping review using a wide range of resources, such as literature and website searches. The accompanying questionnaire survey clarified the current status and background of salt-reduction reformulation efforts, providing valuable information that could not have been obtained through the scoping review alone. However, this study has several limitations. First, the results cannot be generalized due to the limited availability of publicly disclosed information on corporate salt-reduction initiatives. Furthermore, some companies with good practices may have been unintentionally omitted despite a cross-checking process. The questionnaire survey’s findings also lacked generalizability, as only one company and two trade associations participated. Second, there is potential for selection bias. The website search was conducted in English, based on information disclosed on specific sites and from particular regions. Therefore, our findings cannot be directly generalized to companies that do not disclose their commitments, or to companies that disclose commitments in languages other than English. In addition, the companies that responded to the questionnaire were selected through the researchers’ professional networks. Third, this study did not identify what constitutes effective salt-reduction targets. A previous simulation study estimated that population-level salt intake could decrease by 20–27% (1.4–1.8 g/day) or 26–32% (1.8–2.2 g/day) if companies aligned their product reformulation with the 6 g/day or 5 g/day salt intake targets, respectively [81]. In addition to such simulations, future meta-analyses could help clarify the relationship between salt-reduction targets and health outcomes by synthesizing available evidence. Finally, respondents to the survey may have been constrained by internal company policies or institutional interests, which could have influenced their responses.

#### Perspective of Asia

The findings highlight the need for collaboration between governments and companies. In Asia, high sodium intake remains a major public health concern, with estimated average salt consumption in 2030 of 9.7 g/day in the South-East Asia Region (SEAR) and 15.3 g/day in the Western Pacific Region (WPR) [7]. Several countries (11 in SEAR and 27 in WPR) have implemented mandatory and/or voluntary reformulation policies to reduce the sodium content of foods [7]. Through collaboration between governments and companies, clinical guidelines could introduce practical reformulated food options to support healthier food choices among consumers with high blood pressure.

## Conclusion

The companies included in this study established achievable salt-reduction targets by considering government policies and global trends. The findings underscore the importance of multilevel collaboration among government agencies, companies, and other relevant stakeholders to implement sustainable salt-reduction practices. Voluntary reformulation efforts play a vital role in reducing population-level salt intake and contribute meaningfully to global public health and nutrition. This study provides a valuable baseline for promoting company-led salt-reduction initiatives and supporting broader public health goals, particularly the prevention and control of hypertension and cardiovascular disease.

## Supplementary information

Supplementary information
